# Operative versus nonoperative management of olecranon fractures in older adults: a systematic review and meta-analysis

**DOI:** 10.1007/s00402-026-06553-4

**Published:** 2026-11-09

**Authors:** João Pedro Campos Lima, Vicente Morales Ribeiro, Rangel Segatti, Bruno Butturi Varone, Fabrizio dos Santos Cardoso, Vincenzo Giordano

**Affiliations:** 1 Department of Medicine, Afya Centro Universitário Itaperuna, Itaperuna, Brazil; 2 Department of Research, Hospital São Vicente de Paulo, Bom Jesus do Itabapoana, Brazil; 3 Department of Medicine, Universidade do Sul de Santa Catarina, Palhoça, Brazil; 4 Department of Orthopedics and Traumatology, Governador Celso Ramos Hospital, Florianópolis, Brazil; 5 Institute of Orthopedics and Traumatology, Federal University of São Paulo, São Paulo, Brazil; 6 Department of Orthopedics and Traumatology Prof. Nova Monteiro, Hospital Municipal Miguel Couto, Rio de Janeiro, Brazil

**Keywords:** Olecranon fracture, Older adults, Surgery, Nonoperative management, Nonunion

## Abstract

**Introduction:**

Displaced olecranon fractures in the frail geriatric patient represent a therapeutic challenge, involving the decision between operating to achieve anatomical reduction of the articular surface or not operating due to the high risks associated with poor bone quality and failure of mechanical fixation.

**Materials and methods:**

PubMed, Embase, and Cochrane were systematically searched for studies published from inception through February 2026 comparing tension band wiring, plate fixation, or cerclage wiring with nonoperative treatment for displaced olecranon fractures in geriatric patients. Risk ratio (RR) was used to evaluate binary outcomes, and standard mean difference (SMD) for continuous outcomes, with 95% confidence intervals (CIs).

**Results:**

Of 459 articles, eight studies were included. A total of 723 older adults were included. Most patients had Mayo type IIA or IIB fractures. Tension band wiring was the most frequently performed surgical technique, followed by plate fixation and cerclage wiring. Operative treatment was associated with a lower risk of radiographic nonunion (RR 0.19; 95% CI 0.04, 0.8) but increased the risk of overall complications (RR 3.21; 95% CI 1.63, 6.35) and reoperation (RR 4.85; 95% CI 1.72, 13.65) compared to nonoperative treatment. Physical function showed a non-significant trend toward improvement with operative treatment (SMD −0.29; 95% CI -0.72, 0.14). At final follow-up, pain intensity was low and comparable between the two treatment groups across the majority of studies. Approximately half of the reoperation excess comprised elective or symptomatic implant removal, and the overall certainty of the evidence is low.

**Conclusion:**

In older adults with displaced olecranon fractures, operative management was associated with a reduced risk of radiographic nonunion and with a higher risk of complications and reoperation compared to nonoperative management.

**Level of evidence:**

Level III Systematic Review/MetaAnalysis Treatment Study.

**Graphical abstract:**

**Figure:**
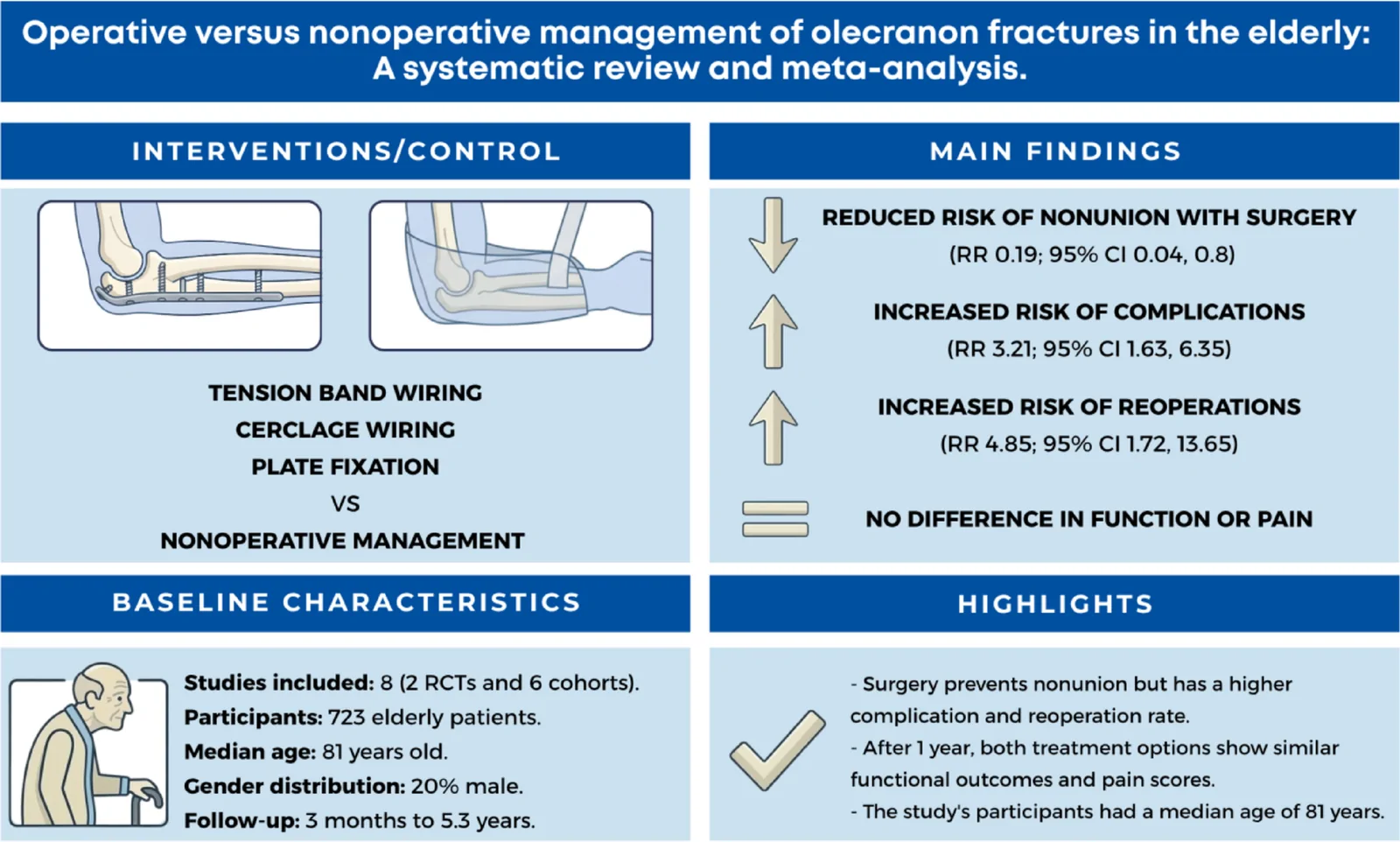

**Supplementary Information:**

The online version contains supplementary material available at https://doi.org/10.1007/s00402-026-06553-4.

## Introduction

Olecranon fractures constitute a significant proportion of upper extremity injuries in geriatric patients, a demographic marked by decreased bone mineral density and susceptibility to low-energy trauma and falls [1, 2]. Annually, these fractures occur in approximately 33 per 100,000 individuals, with incidence increasing after age 50, underscoring their prevalence and the need for effective management strategies [3]. While anatomical restoration of both the extensor mechanism and the articular surface through anatomical reduction and internal fixation, typically tension band wiring or plating, remains the standard of care for younger patients, the optimal management for the geriatric population is increasingly controversial [4]. Surgical intervention in this cohort is frequently associated with substantial morbidity, including hardware prominence, skin problems, fracture-related infection, and high reoperation rates [5].

In contrast to operative fixation, nonoperative management, involving brief immobilization and analgesia, followed by permissive early mobilization, has emerged as a viable alternative [6–8]. This approach values avoiding surgical complications over radiographic union, with recent evidence suggesting that fibrous nonunion often yields satisfactory functional outcomes in lower-demand patients [5].

However, high-level evidence consolidating these competing treatment strategies remains limited in the geriatric population, leaving open questions about their indications and limitations. To date, no dedicated pairwise meta-analysis has directly compared operative and nonoperative management of displaced olecranon fractures in the geriatric population. This study aimed to address this gap by conducting the first pairwise meta-analysis on this topic, systematically appraising and synthesizing available evidence comparing operative and nonoperative management in older adults with displaced olecranon fractures.

## Methods

This systematic review and meta-analysis were conducted in accordance with the Preferred Reporting Items for Systematic Reviews and Meta-Analyses (PRISMA) guidelines [9]. The protocol was registered in PROSPERO (CRD420261323552).

### Eligibility criteria

Eligible studies were randomized controlled trials (RCTs), observational cohort or case-control studies involving older adults (≥ 60 years old) with acute, isolated, displaced olecranon fractures. Studies were required to compare operative treatment with nonoperative management. Operative procedures included tension band wiring, plate fixation, or cerclage wiring, whereas nonoperative management typically consisted of a brief period of immobilization using a sling or cast and analgesia, followed by early range-of-motion exercises as tolerated. The primary outcomes were nonunion, complications, and reoperations. The complete definitions are available in the Supplementary Materials (S2). Secondary outcomes included physical function and pain intensity.

Studies were excluded if they were biomechanical or cadaveric, focused on pathological or open fractures, or failed to report outcomes of interest.

### Search strategy

PubMed, Embase, and the Cochrane Central Register of Controlled Trials (CENTRAL) were searched. The systematic search was conducted from inception through February 2026. The search strategy is available in Supplementary Materials (S1). Restrictions regarding language or publication date were not applied.

### Data extraction

Two review authors independently screened titles and abstracts of identified records to assess eligibility. Full-text articles were obtained for all records that appeared to meet the inclusion criteria or where eligibility was unclear. Any disagreements regarding study selection were resolved through discussion or by consultation with a third review author. Two review authors independently extracted data using a standardized form. Information was collected on study characteristics (design, sample size, follow-up duration), participant demographics (age, sex, comorbidities), intervention details (surgical technique, immobilization protocol), and outcome data. Study authors were contacted to request missing data when necessary.

### Data synthesis

Data were pooled using a random-effects model with the Restricted Maximum Likelihood (REML) estimator, given anticipated clinical and methodological heterogeneity across studies, particularly regarding surgical techniques and rehabilitation protocols. Statistical heterogeneity was assessed using the Chi-squared test and the I2 statistic. An I2 value greater than 50% was considered to represent substantial heterogeneity. For dichotomous outcomes, the risk ratio (RR) with 95% confidence intervals (CIs) was calculated. For continuous outcomes, the standardized mean difference (SMD) with 95% CIs was calculated. A narrative synthesis was performed for outcomes with heterogeneity in statistical reports and in the absence of aggregate data. Leave-one-out plots were used to identify influential studies and their effect on the pooled estimates, evaluating heterogeneity in primary outcomes. This procedure was carried out by sequentially excluding data from individual studies and reanalyzing the remaining dataset to obtain outcomes with at least 3 studies. Statistical analysis was performed using R Software, version 4.2.3.

### Subgroup analysis

To explore the potential influence of follow-up duration on the pooled estimates, subgroup analyses were conducted by follow-up duration (< 12 months versus ≥ 12 months). Studies were categorized by minimum follow-up duration, and subgroup analyses were conducted for all outcomes included in the quantitative synthesis when sufficient data were available.

### Quality assessment

Risk of bias in RCTs was assessed using the Cochrane Risk of Bias 2 (RoB 2) tool [10]. Bias was evaluated across five domains: the randomization process, deviations from intended interventions, missing outcome data, measurement of the outcome, and selection of the reported result. For non-randomized studies, the risk of bias in the Non-randomized Studies - of Interventions (ROBINS-I) tool was used [11]. Bias was assessed across seven domains: confounding, selection of participants into the study, classification of interventions, deviations from intended interventions, missing data, measurement of outcomes, and selection of the reported result. The overall risk of bias for each study was classified as low, moderate, serious, or critical based on judgments across individual domains. Funnel plots were not formally analyzed because fewer than 10 studies were included per outcome, aligned with Cochrane guidance. Exploratory funnel plots are provided in the Supplementary Data for visual inspection.

## Results

**Fig. 1:**
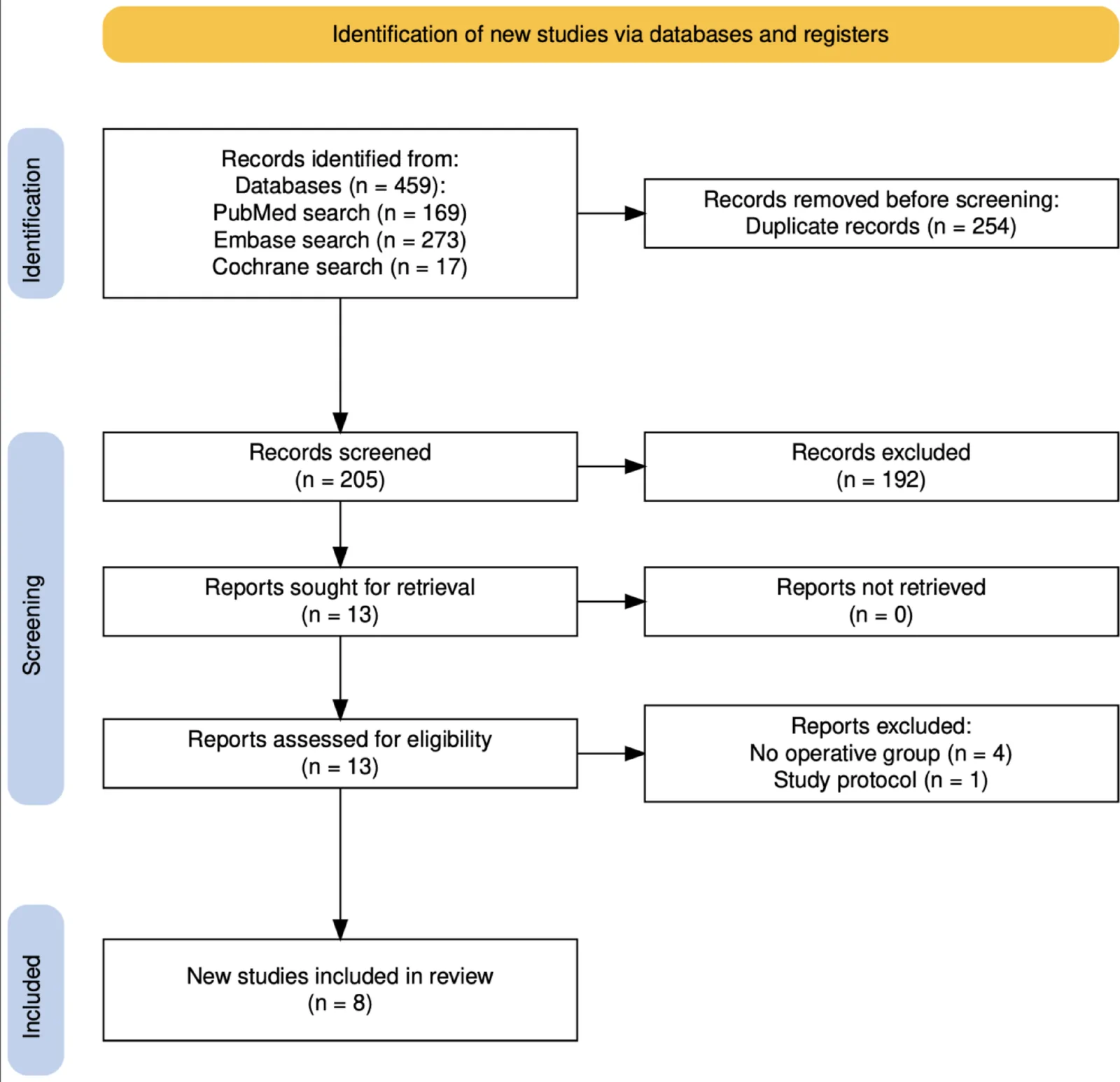
PRISMA flow diagram of study selection

The initial search identified 459 records. After removing 254 duplicates, 205 titles and abstracts were screened for relevance. Of these, 13 full-text articles were assessed for eligibility according to the predefined inclusion criteria. Ultimately, 8 studies [12–19] met the inclusion criteria and were included in the synthesis. The study selection process, including reasons for exclusion [6–8, 20, 21], is detailed in Fig. 1.

The included studies comprised two RCTs and six observational studies, published between 2016 and 2026. The total pooled sample comprised 723 older adults with displaced olecranon fractures, of whom 510 were allocated to the operative management group and 213 to the nonoperative management group. Surgical interventions varied across the included studies, with tension band wiring as the primary fixation method in at least 188 patients, followed by cerclage wiring in 158 patients, plate fixation in 150 patients, 27 patients had an undetermined technique, and one patient received a lag screw; other techniques were used depending on fracture morphology and bone quality. Follow-up duration varied across studies, ranging from 3 months to 5.2 years. A detailed summary of the characteristics of the included studies is presented in Table 1.

**Table 1:** Table 1

Author	Year	Design	Follow-up (minimum or median)	Sample size	Age (mean or median)	Male	Type of fracture	Surgical technique	Immobilization protocol	
Batten	2016	RC	6 months	60	62.62 years	NR	Mayo Type II	TBW (36), Plate (14)	NR	
Duckworth	2017	RCT	12 months	19	83 years	10.5%	Mayo Type IIA & IIB (Displaced ≥ 2 mm)	TBW (9), Plate (2)	Sling or cast for minimum of 2 weeks.	
Joshi	2025	RCT	12 months	60	82.45 years	25%	Mayo Type IIA & IIB (Displaced ≥ 2 mm)	TBW or Plate (27)	Sling for comfort.	
Kaiser	2022	RC	12 months	17	79 years	23.5%	Mayo Type IIA & IIB (Displaced ≥ 5 mm)	Plate (9), TBW (2)	Splint for 5 weeks.	
Luppi	2025	RC	4 months	40	84.5 years	2.5%	Mayo Type IIA & IIB (Displaced ≥ 2 mm)	TBW (20), Lag screw (1)	Cast or brace for a mean of 4 weeks.	
Parkes	2022	RC	4 months	176	80.8 years	28.4%	Mayo Type IA, IB, IIA, IIB, IIIA & IIIC	TBW (82), ORIF (50)	NR	
Wenger	2022	RC	5.2 years	239	81.56 years	13%	Mayo Type IIA & IIB (Displaced ≥ 2 mm)	Cerclage (158), TBW (33), Plate (7)	Cast for 3 weeks.	
Woolnough	2026	RC	3 months	113	81 years	19%	Mayo Type IIA & IIB (Displaced ≥ 5 mm)	Plate (68), TBW (6)	Splint for minimum of 1 week and maximum of 6 weeks.	
Batten et al. included 74 patients overall; 60 (36 TBW and 24 nonoperative) contributed to the comparative analysis; one patient in Duckworth et al. died before follow-up;* RC* Retrospective cohort,* RCT* Randomized controlled trial, *NR* Not reported *TBW* Tension band wiring, *ORIF* Open reduction and internal fixation.										

### Nonunion

**Fig. 2:**
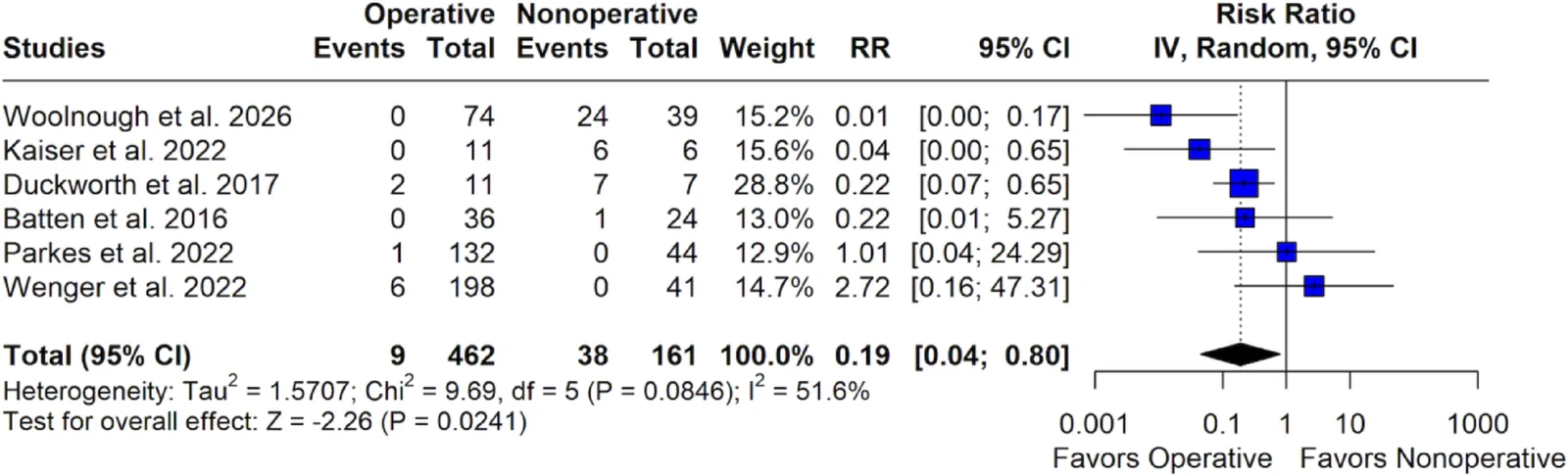
Forest plot comparing radiographic nonunion between operative and nonoperative management

The analysis of radiographic nonunion rates included data from 6 studies [12, 13, 15, 17–19], totaling 623 patients. The synthesis demonstrated that operative intervention was associated with a statistically significant reduction in the risk of nonunion compared to nonoperative management (RR 0.19; 95% CI 0.04, 0.8; *p* < 0.05; I2 = 51%; Fig. 2).

### Complications

**Fig. 3:**
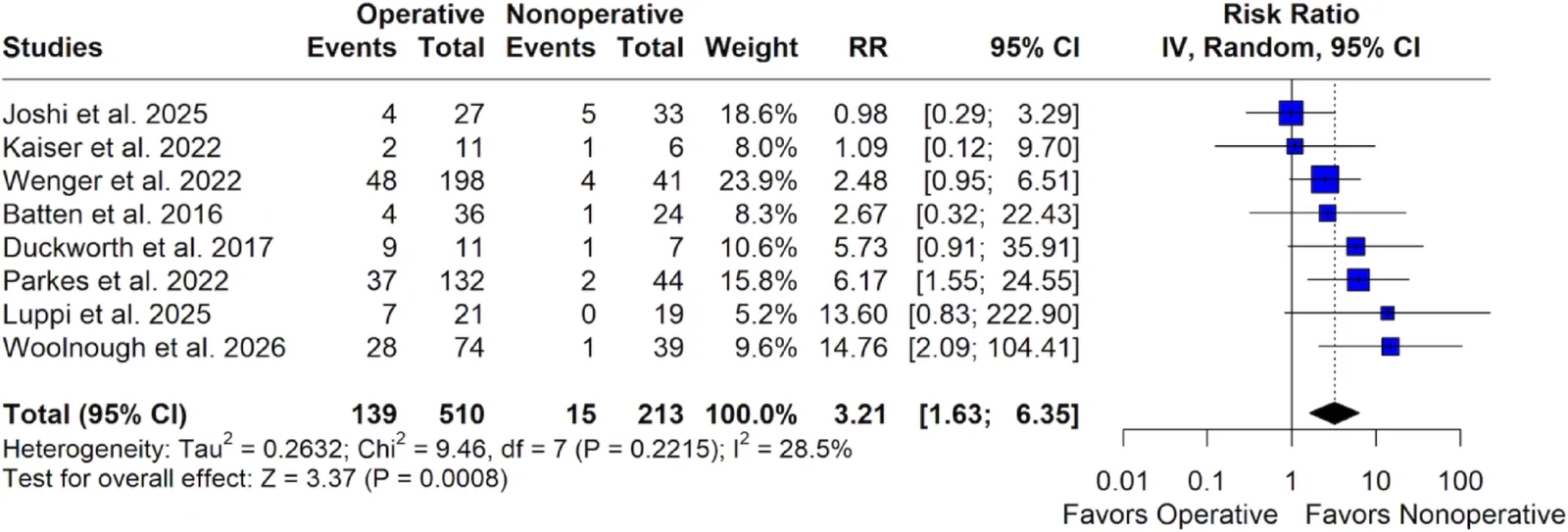
Forest plot comparing overall complications between operative and nonoperative management

All 8 studies [12–19] reported complications (510 operated patients and 213 nonoperated patients). The pooled analysis indicated a statistically significant increase in the risk of complications among surgically treated patients compared with those managed nonoperatively (RR 3.21; 95% CI 1.63, 6.35; *p* < 0.05; I2 = 28%; Fig. 3). The incidence of complications is described in the Supplementary Material, when available, in the articles analyzed.

### Reoperation

**Fig. 4:**
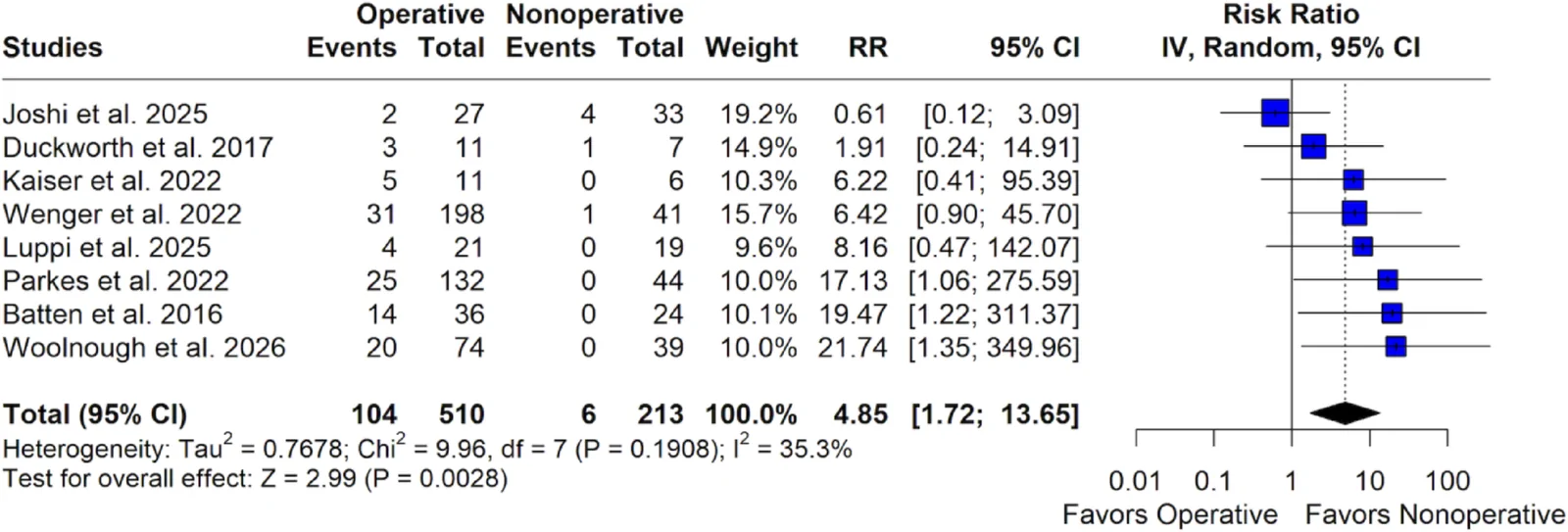
Forest plot comparing reoperation rates between operative and nonoperative management

Reoperation rates were reported in all studies [12–19], involving the same cohort of 723 patients. The operative group had a significantly higher risk of requiring subsequent surgical procedures than the nonoperative group (RR 4.85; 95% CI 1.72, 13.65; *p* < 0.05; I2 = 35%; Fig. 4).

### Physical function

**Fig. 5:**
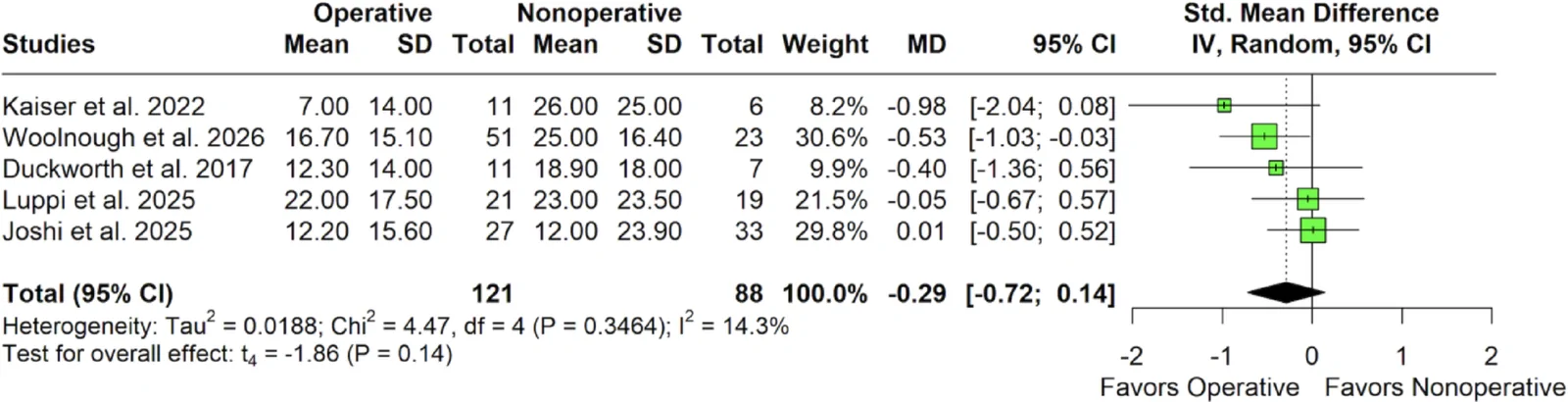
Forest plot comparing physical function between operative and nonoperative management

Upper limb function was evaluated using the Disabilities of the Arm, Shoulder, and Hand (DASH) score or its shortened version (QuickDASH), recorded at the final follow-up assessment. These validated 100-point scales are scored inversely, with lower values indicating superior functional performance and reduced disability. Constrained by the low sample size of the included studies, the pooled estimate demonstrated a trend toward improved functional outcomes in the operative group compared to nonoperative treatment, although this difference did not reach statistical significance (SMD −0.29; 95% CI −0.72, 0.14; *p* = 0.14; I2 = 14%; Fig. 5).

### Pain intensity

Due to heterogeneity in assessment methods and statistical reporting, and the lack of aggregate data, it was not possible to perform a quantitative meta-analysis. Therefore, a narrative synthesis was performed to assess pain intensity. Five studies [12–16] evaluated pain severity using the Visual Analog Scale (VAS), consistently reporting low to negligible long-term pain levels regardless of treatment allocation. Both RCTs found no significant difference in pain scores at 12 months. Joshi et al. [14] reported mean VAS scores of 0.9 ± 1.5 for the operative group and 1.2 ± 1.9 for the nonoperative group (*p* = 0.45). Similarly, Duckworth et al. [13] reported a median pain score of 0 (range 0–6) for both cohorts, noting that the development of fibrous non-union was not associated with residual pain.

Retrospective cohort studies corroborated these findings. Luppi et al. [16] observed no significant difference in mean pain scores between the operative and nonoperative groups (1.56 vs. 1.0; *p* = 0.39) at a mean follow-up of over three years. Kaiser et al. [15] found that while resting pain was 0 for all patients, pain under load was comparable between the operative and nonoperative groups (0.5 ± 1.2 vs. 1.0 ± 1.3; *p* = 0.35). In contrast, Batten et al. [12] identified a subgroup of patients aged > 74 years with 5–10 mm of displacement who reported significantly higher pain following operative fixation compared to nonoperative management (4.75 vs. 1.28; *p* = 0.007), a finding the authors attributed to symptomatic hardware.

## Subgroup analyses

### Nonunion

The pooled effect favored operative treatment in both follow-up subgroups. In studies with follow-up < 12 months, the pooled RR was 0.12 (95% CI 0.01, 1.79; I² = 57.8%), whereas in studies with follow-up ≥ 12 months, the pooled RR was 0.27 (95% CI 0.04, 1.70; I² = 53.8%). No significant subgroup difference was observed (*p* = 0.64; Supplementary Fig. 3).

### Complications

Operative treatment was associated with a higher risk of complications in both follow-up subgroups. The pooled RR was 6.44 (95% CI 2.37, 17.45; I² = 0%) in studies with follow-up < 12 months and 2.18 (95% CI 1.09, 4.37; I² = 17.5%) in studies with follow-up ≥ 12 months. No significant subgroup difference was detected (*p* = 0.08; Supplementary Fig. 4).

### Reoperation

Operative treatment remained associated with a higher risk of reoperation regardless of follow-up duration. However, the effect estimate was greater in studies with follow-up < 12 months (RR 15.74; 95% CI 3.89, 63.69; I² = 0%) than in those with follow-up ≥ 12 months (RR 2.17; 95% CI 0.64, 7.39; I² = 26.5%). A statistically significant subgroup difference was identified (*p* = 0.04; Supplementary Fig. 5).

Because a substantial proportion of the events recorded after fixation cannot occur in the absence of an implant, complications and reoperations were additionally tabulated by whether the event was specific to operative treatment (Supplementary Tables S15 and S16).

### Physical function

No significant differences in physical function were observed in either follow-up subgroup. The pooled SMD was −0.31 (95% CI −1.50, 0.88; I² = 30.7%) in studies with follow-up < 12 months and −0.33 (95% CI −3.35, 2.70; I² = 28.8%) in studies with follow-up ≥ 12 months. No significant subgroup difference was found (*p* = 0.96; Supplementary Fig. 6).

### Sensitivity analysis

**Fig. 6:**
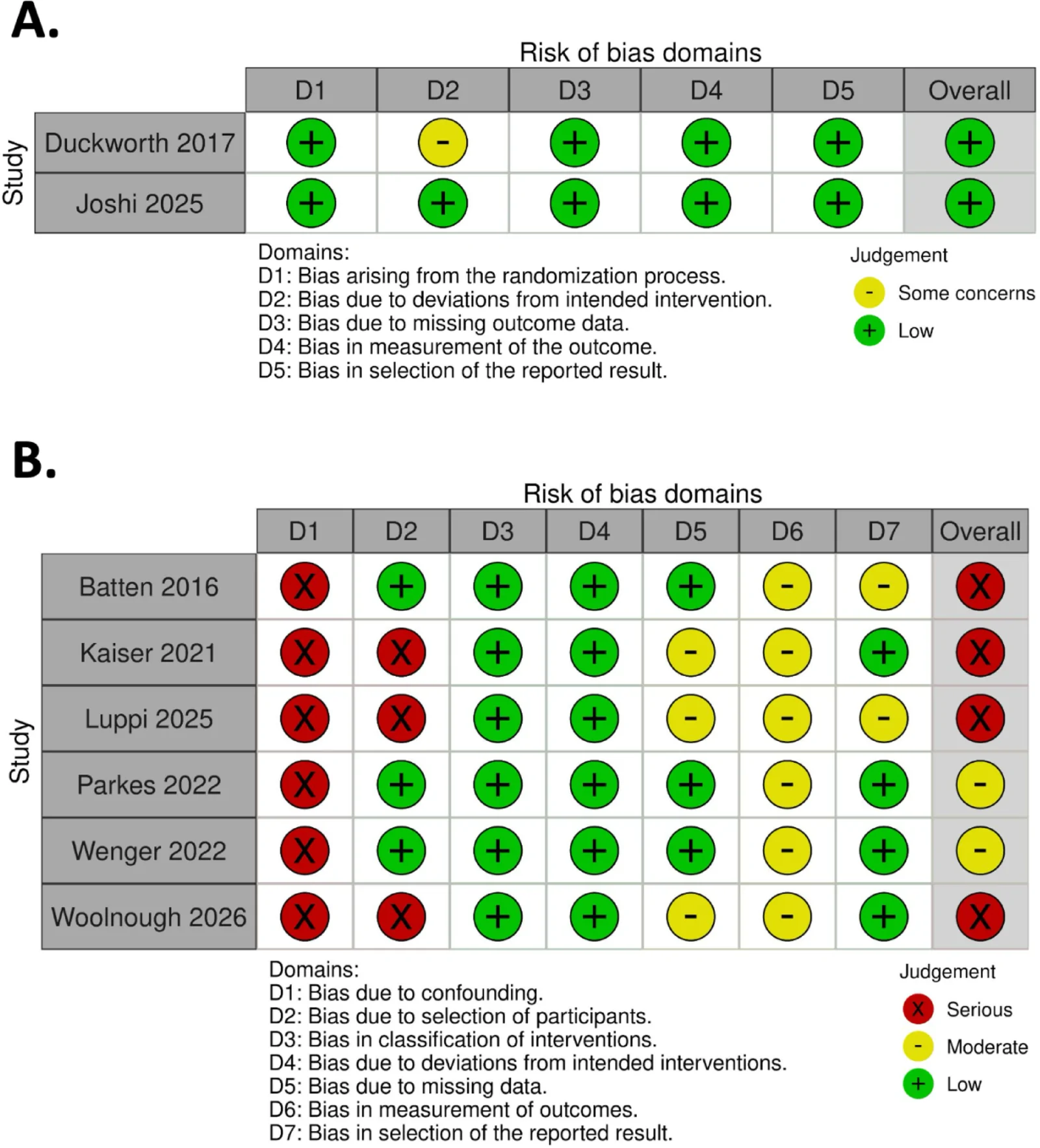
Risk of bias assessment of the included studies. **A** Risk of bias assessment of randomized controlled trials using the RoB 2 tool. **B** Risk of bias assessment of non-randomized studies using the ROBINS-I tool

Leave-one-out sensitivity analysis for primary outcomes demonstrated consistency in results for complications and reoperations. For nonunion, omitting Woolnough et al. [19] reduced heterogeneity, likely because it was the study that most frequently used plate fixation. However, omitting Batten et al. [12], Duckworth et al. [13], or Kaiser et al. [15] did not yield a significant difference between the groups. These findings suggest a potential lack of benefit for surgically treated patients compared with nonoperative treatment in terms of nonunion. Sensitivity analyses are presented in the Supplementary Materials (S7-S10) Fig. 6.

### Risk of bias assessment

Overall, the RCTs were judged to be at low risk of bias for the primary outcome. Randomization and allocation procedures were adequate, resulting in balanced baseline characteristics, and missing outcome data were minimal, suggesting minimal attrition bias. Outcome assessment relied on standardized, objective radiographic criteria, and selective reporting was considered unlikely given prospective trial registration and the availability of published protocols. One trial was judged to have some concerns regarding blinding. However, as nonunion is an objective radiographic outcome, the absence of blinding was unlikely to have influenced the effect estimates. Another trial reported appropriate blinding of outcome assessors and the statistician, with minimal crossover, supporting a low risk of bias in this domain.

In contrast, non-randomized studies showed greater methodological limitations. Two studies were assessed as having a moderate risk of bias, while four were judged to have a serious risk of bias. Overall, all studies were downgraded primarily because of their retrospective design and inadequate control for important confounding factors, resulting in a serious risk of bias. Additional concerns emerged about patient exclusions from the final analyses, most commonly due to death, without appropriate adjustment for selection bias, posing a serious risk of bias. Consequently, these exclusions raised concerns about missing outcome data and resulted in further downgrading.

By contrast, the definition and implementation of the interventions were clear and consistent across studies, supporting a low risk of bias related to intervention classification and deviations from intended interventions. The measurement of results was considered reliable because the primary outcomes were objective and clearly defined, but due to the lack of blinding, the risk of bias in the assessment of results was moderate. Finally, reporting-related limitations were identified in two studies, one due to inadequate subgroup analyses and the other due to high exclusion rates after participant selection.

## Discussion

This systematic review and meta-analysis compared operative versus nonoperative management of displaced olecranon fractures in older adults (median age of 81 years), synthesizing evidence from eight studies comprising 723 participants. Operative fixation was associated with a lower risk of radiographic nonunion in the primary analysis. However, this finding was not robust across sensitivity and follow-up subgroup analyses. This radiographic advantage came at the expense of significantly higher complication and reoperation rates. In clinically meaningful terms, operative treatment achieved an absolute risk reduction (ARR) of 21.7% for nonunion compared with nonoperative management, corresponding to a number needed to treat (NNT) of 5 to prevent one additional nonunion. On the other hand, nonoperative treatment produced an ARR of 20.3% for overall complications and 17.6% for reoperations relative to surgery, translating into NNTs of 5 and 6, respectively, to prevent one additional complication or reoperation. There was no statistically significant difference in upper-limb function, and long-term pain remained consistently low in both groups.

This evidence indicates that in geriatric patients, the benefit of surgery in achieving radiographic anatomical articular reduction and union is modest, considering the significantly higher rates of complications and reoperations. From a patient-centered perspective, the balance of benefits and harms favors nonoperative management, as the radiographic advantage of surgery does not clearly translate into superior patient-reported outcomes. These results support a treatment paradigm that favors avoiding surgical morbidity over maximizing the likelihood of bone union in this population.

Supporting this perspective, interventional studies [6–8, 20, 22, 23] without control groups have reported high patient satisfaction and satisfactory functional outcomes even when radiographic nonunion rates exceed 70%. Importantly, our definition of radiographic nonunion included fibrous union and fibrous nonunion. Thus, the higher radiographic nonunion rate observed after nonoperative treatment should not necessarily be interpreted as treatment failure, as a stable fibrous union may be well tolerated and does not necessarily compromise daily independence, despite some expected weakness and limitation in elbow flexion and extension [13, 23, 24]. Of interest, there is no standard rehabilitation protocol for the nonoperative treatment of these injuries in older adults [8, 15, 20, 24, 25]. Although this was not the objective of this study, we believe some of these potential deficits may result from the lack of a uniform, reproducible physical therapy strategy. By contrast, operative interventions are more frequently associated with hardware-related complications, reoperations, and no significant survival advantage [6, 17].

The high complication rates with operative management are, in part, biologically plausible. Histological evidence [26] demonstrated a significant age-related deterioration in bone microarchitecture, specifically a 45% reduction in trabecular meshwork volume in patients aged 65 years or older. As a result, this osteopenic environment compromises the quality of both the reduction, since articular impaction and comminution in olecranon fractures have been associated with worse postoperative outcomes [27, 28], and the fixation, increasing the risk of hardware loosening [28]. This suggests that the low age-related bone attenuation observed in geriatric patients with olecranon fractures substantially increases the risk of reoperation [2, 28], as observed in our study.

These observations are further supported by subgroup analyses stratified by follow-up duration. The effect observed for reoperations in studies with shorter follow-up is clinically plausible, as secondary procedures after olecranon fixation, particularly for symptomatic hardware, fixation failure, and early wound complications, typically occur within the first postoperative year, suggesting that the excess risk associated with operative treatment is concentrated early in the postoperative course rather than accumulating progressively over time. This inference should nevertheless be interpreted with caution, because it rests on comparisons across studies rather than on longitudinal changes within a single cohort, and may also reflect differences in study design, patient characteristics, and surgical practice.

Additionally, the composition of these procedures influences the magnitude of the effect just as much as its timing. Elective or symptomatic implant removal accounted for approximately half of the reoperations recorded in the operative group and has no counterpart in the nonoperative arm, so part of the observed excess is definitional rather than a difference in treatment failure. It does not, however, account for the whole of the effect: reoperation for fixation failure, infection, nonunion, or crossover alone remained more frequent after fixation.

Regarding functional assessment, Franchignoni et al. [29] established the minimal clinically important difference (MCID) for DASH and QuickDASH as 10.83 and 15.91 points, respectively. In the present analysis, the difference in physical function between groups did not reach statistical significance, likely due to the small sample size. Furthermore, although using SMD is not ideal because of the variety of scales, the observed differences are below the MCID threshold, suggesting limited clinical relevance.

In real-world clinical practice, treatment preferences vary considerably. A cross-sectional survey indicated that surgeons tend to favor nonoperative management for patients over 75 years of age and for those with comminuted fractures [30]. This highlights the role of clinical judgment and patient frailty in decision-making, often taking precedence over strict adherence to radiographic criteria. Surgical intervention may be indicated for unstable fractures, fracture-dislocations, or in more active older adults, always considering a higher risk of reoperation and hardware-related complications, particularly in certain conditions such as osteoporotic bone, inadequate soft-tissue envelope, and poor adherence to the postoperative regimen [5, 17, 31].

A cost-minimization analysis by Welch et al. [32], using a United States Medicare claims database of 2,433 geriatric patients, demonstrated that the mean total cost over 1 year was US$10,694 for operative management versus US$2,544 for nonoperative management. Based on these estimates, if nonoperative treatment were adopted as the standard of care for low-demand patients, consistent with the functional equivalence demonstrated in the present analysis, treating 100 such patients could hypothetically be associated with an estimated saving of approximately US$815,000, excluding indirect costs such as productivity losses, caregiver burden, and direct patient expenses. However, this estimate should be interpreted with caution because it is derived from a single United States Medicare database and may not be generalizable to other healthcare systems with different reimbursement models, resource utilization, and cost structures.

Our findings concur with previous reviews. Chen et al. [33] conducted a meta-analysis of proportions in 300 older adults and reported similar results across strategies: reoperation rates of 40% for tension band wiring, 33% for plating, and 0% for nonoperative management. Similarly, Alvara et al. [34] reviewed 69 geriatric patients treated nonoperatively and found that, although only 25% achieved radiographic union, 92% reported a mean DASH score of 16.9, indicating minimal disability, and a complication rate of only 26%, mostly minor pain not requiring intervention. These reviews corroborate our primary finding that functional nonunion is well tolerated in this population, while surgical intervention carries significant hardware-related morbidity and other complications.

Taken together, these findings should be read as low-certainty evidence. Six of the eight studies were retrospective and four were at serious risk of bias, chiefly for uncontrolled confounding by indication, and the confidence intervals were wide for every pooled outcome. The signal is most secure for harms: complications and reoperation were consistent in direction across studies, and the preferential allocation of fitter patients to surgery would, if anything, understate them. It is weakest for radiographic nonunion, which lost statistical significance in both follow-up subgroups and on leave-one-out analysis, and for upper-limb function, where the interval remains compatible with a clinically appreciable advantage of surgery. 

### Limitations

Several limitations must be acknowledged. First, of the eight studies included, only two were randomized controlled trials (RCTs). The predominance of low-quality retrospective observational studies introduces susceptibility to confounding by indication, as healthier, lower-risk patients were likely selected for surgery while frailer patients were allocated to nonoperative care, potentially biasing functional comparisons in favor of the operative group. Second, the inability to blind surgeons and patients to treatment allocation creates a risk of performance and detection bias, particularly for subjective outcomes such as pain and DASH scores. Third, while statistical analysis demonstrated moderate heterogeneity, this likely underestimates the considerable clinical diversity across populations, ages, surgical techniques, frailty, follow-up duration, and postoperative management. In particular, nonoperative treatment protocols varied substantially across studies, ranging from simple sling immobilization to cast immobilization for up to five weeks, which may have influenced functional recovery and other pooled outcomes. Additionally, the eligibility threshold of ≥ 60 years resulted in the inclusion of a comparatively younger population in Batten et al., which may limit the generalizability of our findings to older geriatric populations. Fracture patterns were also heterogeneous, as Parkes et al. included Mayo type I and III fractures in addition to type II fractures, and outcome data stratified by fracture type were unavailable. Technique-specific subgroup meta-analyses could not be reliably performed because outcomes were not consistently reported separately for tension-band wiring, plate fixation, cerclage wiring, and other operative techniques. Finally, the nonoperative group had a relatively small sample size, especially for the functional and pain intensity outcomes. 

## Conclusions

It is also important to highlight the strengths of this study. First, it represents the first dedicated pairwise meta-analysis to directly compare the two treatment options in this population, covering a critical gap left by prior reviews that relied on indirect comparisons or single-arm studies. The internal validity of these results is strengthened by the inclusion of recent studies, particularly the Level I evidence from the SOFIE trial [14]. Furthermore, the consistency of results is supported by the acceptable statistical heterogeneity across all primary outcomes, suggesting a stable treatment effect despite variation in healthcare settings and surgical techniques. Finally, our results show acceptable functional outcomes and low rates of functional complications and residual pain, which are consistent with the observation that non-surgical treatment is not inferior to surgical treatment, as already demonstrated in other articular fractures of the upper limb in the geriatric population [35–37].

The available evidence suggests that, for older adults with displaced olecranon fractures, nonoperative management is associated with fewer complications and reoperations, without impairing long-term function or pain control, compared with operative fixation. This suggests that nonoperative management may be a preferable option for low-demand geriatric patients, prioritizing avoidance of surgical morbidity over radiographic union, although eligibility in the included studies was defined principally by chronological age, frailty was formally quantified in only one cohort [19], in which it was a stronger determinant of limb-specific disability than age, and functional demand was not measured in any, so that low demand could not be examined as an effect modifier in the pooled analysis. Future high-quality RCTs are needed to confirm the findings.

## Supplementary Information

Below is the link to the electronic supplementary material.

Supplementary Material 1

## Data Availability

No datasets were generated or analysed during the current study.
