# Supplementary material for: Operative versus nonoperative management of olecranon fractures in older adults: a systematic review and meta-analysis
Source: Arch Orthop Trauma Surg. 2026 Nov 9;146(1):401. doi: 10.1007/s00402-026-06553-4 (PMC13748076; doi:10.1007/s00402-026-06553-4)
Supplement: Supplementary file 1 [file 402_2026_6553_MOESM1_ESM.docx]

***SUPPLEMENTARY MATERIAL***

**Operative versus nonoperative management of olecranon fractures in older adults:
A systematic review and meta-analysis.**

**Table of Contents**

**Supplementary Table S1. Search strategies for each database .......... 3**

**Supplementary Material S2. Primary outcomes definitions .......... 4**

**Supplementary Figure S3. Nonunion subgroup analysis .......... 5**

**Supplementary Figure S4. Complications subgroup analysis .......... 5**

**Supplementary Figure S5. Reoperations subgroup analysis .......... 6**

**Supplementary Figure S6. Physical function subgroup analysis .......... 6**

**Supplementary Figure S7. Leave-one-out analysis for nonunion .......... 7**

**Supplementary Figure S8. Leave-one-out analysis for complications .......... 7**

**Supplementary Figure S9. Leave-one-out analysis for reoperations .......... 7**

**Supplementary Figure S10. Leave-one-out analysis for physical function .......... 8**

**Supplementary Figure S11. Funnel plot for nonunion outcome .......... 9**

**Supplementary Figure S12. Funnel plot for complications outcome ......... 9**

**Supplementary Figure S13. Funnel plot for reoperations outcome ......... 10**

**Supplementary Figure S14. Funnel plot for function outcome ......... 10**

**Supplementary Table S15. Complications frequency analysis ......... 11**

**Supplementary Table S16. Reoperation events by indication .........11**

**Supplementary Table S1. Search strategies for each database.**

**PubMed**

(olecranon OR "proximal ulna") AND (fracture*) AND (surgery OR surgical OR operative OR "tension band" OR plate OR fixation OR wiring) AND (nonoperative OR non-operative OR conservative OR non-surgical)

**Embase**

(olecranon OR "proximal ulna") AND (fracture*) AND (surgery OR surgical OR operative OR "tension band" OR plate OR fixation OR wiring) AND (nonoperative OR non-operative OR conservative OR non-surgical)

**Cochrane**

(olecranon OR "proximal ulna") AND (fracture*) AND (surgery OR surgical OR operative OR "tension band" OR plate OR fixation OR wiring) AND (nonoperative OR non-operative OR conservative OR non-surgical)

**●	PubMed: 169 records**

**●	Embase: 273 records**

**●	Cochrane Library: 17 records**

**●	Total: 459 records identified**

**Supplementary Material S2. Primary outcomes definitions.**

**Primary outcomes definitions**

The primary outcome was nonunion, defined as the absence of radiographic union at the latest reported follow-up for fracture healing, operationalized as failure to achieve at least 75% organized trabecular bridging across the fracture site on anteroposterior and lateral radiographs. Any case explicitly described by the study authors as nonunion, fibrous nonunion, fibrous union, or pseudarthrosis was classified as nonunion under this definition.

Complications were defined as any event explicitly reported by the authors as an adverse event or complication, or as a deviation from the expected clinical course attributable to the fracture or its management. This included, but was not limited to, fixation failure, loss of reduction, hardware loosening, wound-related problems, superficial or deep infection, neurological symptoms, or symptomatic hardware.

Reoperation was defined as any unplanned surgical procedure performed after the initial treatment of the index fracture, including revision fixation, surgical debridement for infection, hardware removal for symptomatic implants, or conversion from nonoperative management to operative intervention.

**Supplementary Figure S3. Nonunion subgroup analysis**

**Supplementary Figure S4. Complications subgroup analysis**

**Supplementary Figure S5. Reoperations subgroup analysis**

**Supplementary Figure S6. Physical function subgroup analysis**

**Supplementary Figure S7. Leave-one-out analysis for nonunion.**

**Supplementary Figure S8. Leave-one-out analysis for complications.**

**Supplementary Figure S9. Leave-one-out analysis for reoperations.**

 **Supplementary Figure S10. Leave-one-out analysis for physical function.**

**Supplementary Figure S11. Funnel plot for nonunion outcome.**

**Supplementary Figure S12. Funnel plot for complications outcome.**

**Supplementary Figure S13. Funnel plot for reoperations outcome.**

**Supplementary Figure S14. Funnel plot for function outcome.**

**Supplementary Table S15. Complications frequency analysis.**

| **Study** | **Complication type** | **Occurs only after fixation** | **Nonoperative events** | **Operative events** |
| --- | --- | --- | --- | --- |
| **Batten 2016** | Metalwork or fixation failure | Yes | 0/24 | 3/36 |
|  | Ulnar nerve neuropathy | No | 0/24 | 1/36 |
| **Duckworth 2017** | Loss of reduction | No | 1/7 | 6/11 |
|  | Removal of metalwork | Yes | 0/7 | 3/11 |
| **Joshi 2025** | Deep infection with secondary fixation failure | Yes | 0/33 | 1/27 |
|  | Hardware irritation | Yes | 0/33 | 1/27 |
|  | Hardware migration and fracture displacement | Yes | 0/33 | 1/27 |
|  | Painful nonunion | No | 1/33 | 0/27 |
|  | Skin tenting with progression to open fracture | No | 1/33 | 0/27 |
|  | Crossover to surgery, reason not stated | No | 2/33 | 0/27 |
|  | Death, unrelated | No | 1/33 | 1/27 |
| **Kaiser 2022** | Ulnar nerve palsy | No | 1/6 | 0/11 |
|  | Prolonged lymphedema | No | 0/6 | 1/11 |
|  | Revision for screw blocking forearm rotation | Yes | 0/6 | 1/11 |
| **Luppi 2025** | Prominent symptomatic metalwork | Yes | 0/19 | 4/21 |
|  | Loss of reduction | No | 0/19 | 3/21 |
| **Parkes 2022** | Metalwork loosening alone | Yes | 0/44 | 22/132 |
|  | Loss of reduction with metalwork loosening | Yes | 0/44 | 2/132 |
|  | Loss of reduction alone | No | 0/44 | 1/132 |
|  | Surgical site infection | Yes | 0/44 | 3/132 |
|  | Wound dehiscence | Yes | 0/44 | 3/132 |
|  | Conversion to open reduction and internal fixation | Yes | 0/44 | 1/132 |
|  | Periprosthetic fracture | Yes | 0/44 | 1/132 |
|  | Nonunion | No | 0/44 | 1/132 |
|  | Stiffness | No | 0/44 | 1/132 |
|  | Ipsilateral humeral fracture | No | 1/44 | 0/132 |
| **Wenger 2022** | Local soft-tissue problems | Yes | 0/41 | 26/198 |
|  | Infection | No | 3/41 | 18/198 |
|  | Sensory deficit | No | 1/41 | 1/198 |
| **Woolnough 2026** | Implant irritation | Yes | 0/39 | 10/74 |
|  | Aseptic proximal fragment escape | Yes | 0/39 | 8/74 |
|  | Deep infection | Yes | 0/39 | 4/74 |
|  | Superficial wound complication | Yes | 0/39 | 3/74 |
|  | Transient neuropraxia | Yes | 0/39 | 1/74 |
|  | Frozen shoulder | No | 0/39 | 2/74 |
|  | Cast or splint irritation | No | 1/39 | 0/74 |
| **Subtotal** | Events occurring only after fixation | Yes | 0 | 98 |
| **Subtotal** | Events possible under either treatment | No | 13 | 36 |
| **Total** | All complication events | — | 13 | 134 |

**Supplementary Table S16. Reoperation events by indication.**

| **Study** | **Arm** | **N** | **Elective or symptomatic implant removal** | **Reoperation for fixation failure, infection, nonunion or crossover** | **Indication not itemised** | **Total reoperations** |
| --- | --- | --- | --- | --- | --- | --- |
| **Batten 2016** | Nonoperative | 24 | 0 | 0 | 0 | 0 |
|  | Operative | 36 | 10 | 0 | 4 | 14 |
| **Duckworth 2017** | Nonoperative | 7 | 0 | 1 | 0 | 1 |
|  | Operative | 11 | 3 | 0 | 0 | 3 |
| **Joshi 2025** | Nonoperative | 33 | 0 | 4 | 0 | 4 |
|  | Operative | 27 | 1 | 1 | 0 | 2 |
| **Kaiser 2022** | Nonoperative | 6 | 0 | 0 | 0 | 0 |
|  | Operative | 11 | 4 | 1 | 0 | 5 |
| **Luppi 2025** | Nonoperative | 19 | 0 | 0 | 0 | 0 |
|  | Operative | 21 | 3 | 1 | 0 | 4 |
| **Parkes 2022** | Nonoperative | 44 | 0 | 0 | 0 | 0 |
|  | Operative | 132 | 0 | 25 | 0 | 25 |
| **Wenger 2022** | Nonoperative | 41 | 0 | 1 | 0 | 1 |
|  | Operative | 198 | 21 | 8 | 2 | 31 |
| **Woolnough 2026** | Nonoperative | 39 | 0 | 0 | 0 | 0 |
|  | Operative | 74 | 10 | 10 | 0 | 20 |
| **Total** | Nonoperative | 213 | 0 | 6 | 0 | 6 |
|  | Operative | 510 | 52 | 46 | 6 | 104 |
